# The hydration mechanism of geopolymers based on the activity of solid waste precursors and the evolution of their mechanical properties

**DOI:** 10.1371/journal.pone.0336339

**Published:** 2025-11-12

**Authors:** Miaomiao Gong, Rui He, Yiyi Wang, Ao Shen, Huaiyi Wang, Linghui Sun, Xiaohan Zhan, Jianshe Liu

**Affiliations:** 1 College of Hydraulic and Civil Engineering, Xinjiang Agricultural University, Urumqi, Xinjiang, PR China; 2 Xinjiang Hydraulic Engineering Geotechnical and Structural Engineering Technology Research Center, Urumqi, Xinjiang, PR China; 3 Xinjiang Research Institute of Water Resources and Hydropower, Xinjiang Engineering and Technology Research Center of Water Resources and Hydropower Materials, Urumqi, Xinjiang, China; 4 Xinjiang Production and Construction Corps Survey and Design Institute Group Co., Ltd, Urumqi, Xinjiang, PR China; SASTRA Deemed University, INDIA

## Abstract

Using slag and fly ash as raw materials to prepare geopolymers is an effective approach to achieving high-value utilization of industrial solid waste and reducing the carbon footprint of building materials. In this study, calcium carbide slag and sodium sulfate were used as composite activators to design three geopolymer systems with high, medium, and low activity. Through multi-scale characterization techniques (XRD/SEM/FTIR/TG-DSC/MIP) combined with mechanical property testing, the performance evolution patterns during the aging hardening process were systematically investigated. The results indicate. The high-activity system exhibits intense early hydration reactions, with compressive strength reaching 46.5 MPa at 28 days (87% of the 90-day strength), indicating that time-dependent hardening primarily occurs in the early stages. The low-activity system exhibits a sustained increase in strength, with a strength growth rate of 51.5% (from 23.9 to 36.2 MPa) between 28 and 90 days. Sodium sulfate significantly enhances late-stage performance by promoting the leaching of [AlO_4_]^5-^. The type of activator and the activity of the precursor significantly influence the mechanical properties and hydration process of geopolymers, providing a theoretical basis for optimizing geopolymer formulations.

## 1. Introduction

The accumulation of industrial solid waste worldwide has become a serious environmental challenge. According to statistics from the International Energy Agency, in 2021, coal-fired power plants generated 1.23 billion tons of fly ash, while the steel industry produced approximately 480 million tons of blast furnace slag. China contributed 650 million tons and 290 million tons, respectively [1,2]. The comprehensive utilization rate of these wastes is less than 60%. Large-scale storage not only occupies land (each 10,000 tons of solid waste occupies approximately 1.5 mu of land) but also causes serious heavy metal pollution. At the same time, traditional silicate cement production emits approximately 2.8 billion tons of CO_2_ annually, accounting for 8% of global anthropogenic emissions. (International Cement Review, 2023) In this context, developing geopolymer materials using industrial solid waste as raw materials has dual significance: it can alleviate environmental pressure through the resource utilization of solid waste while also reducing CO_2_ emissions.

The activity of geopolymer precursor materials has a significant impact on their mechanical properties. The activity of slag and fly ash, which are commonly used precursor materials, is mainly affected by their chemical composition, mineral composition and particle characteristics. Highly reactive slag generally contains more glass phase and reactive SiO_2_ and Al_2_O_3_, which, under the action of an activator, help to form a dense geopolymer structure, thus enhancing the material’s mechanical properties. The reactivity of fly ash is closely related to its fineness, loss on ignition and vitreous content. Fly ash with high fineness and low loss on ignition generally exhibits better reactivity. Studies have shown that alkali-activated slag gel exhibits the property of quick hardening and early strength development [3], while alkali-activated Class F fly ash has a significantly different performance from alkali-activated slag gel, as its strength development is relatively slow under normal temperature curing conditions [4,5].

Common alkali activators, such as sodium hydroxide, sodium silicate, and sodium sulfate, are widely used to enhance material properties. Among these, sodium hydroxide and sodium silicate are particularly effective in raising the pH level of the material and promoting the hydration reaction of the mineral powder [6,7]. Sodium silicate, as an activator, can not only increase the alkali equivalent and mineral powder content of the filling material but also reduce the porosity and enhance the strength at all ages [8–10]. However, it may also increase the viscosity of the fresh filling material and affect its fluidity [11]. In contrast, sodium sulfate aqueous solution usually has a pH of 9 to 10, which has a limited effect on improving the hydration ability of precursor materials [12]. However, it can promptly react with calcium hydroxide, a hydration-derived component of Portland cement, resulting in higher alkalinity of the slurry [13]. The dissolution-precipitation process of the sodium sulfate-activated cementitious material usually lasts about 5 days, during which the material can set, harden, and develop a certain strength. In addition, through the testing methods of XRD and NMR, the investigation demonstrated that the mineral powder’s reaction level consistently improved throughout the 18-month curing duration, with C-(A)-S-H and ettringite contents also escalating as curing time advanced [14].

Abdullah et al. [15] conducted a systematic review of the improvement of concrete performance by mineral admixtures such as fly ash, confirming their effectiveness as auxiliary cementitious materials; Ahmad et al. [16] further discovered that the synergistic effect of fly ash and polypropylene fibers can significantly enhance the mechanical properties of foam concrete. Additionally, the combined application of high-strength materials and solid waste provides new insights into the structural application of solid waste-based materials [17].

In high-performance concrete with a mixture of slag and fly ash, early strength decreases with increasing slag content, but the late strength gradually increases with age, especially when the water-to-cement ratio is low. In addition, under the combined action of chloride ions and carbonation, the co-injection of slag and fly ash can improve the pore structure of the concrete and enhance its resistance to chloride ion penetration and carbonation [18].

The mechanical performance and microstructural evolution of sodium sulfate-activated backfill exhibited progressive improvement with prolonged curing duration. During the early curing phase (3–7 days), sodium sulfate significantly enhanced the early strength by promoting ettringite (AFt) formation, reducing free water content, and strengthening interparticle bonding. As curing continued (14–28 days), the pore structure became increasingly compact. While strength development persisted, its growth rate exhibited a gradual deceleration [19].

The aforementioned studies indicate that the activity of precursor materials and their interaction with activators have a decisive influence on the mechanical properties of geopolymers. Current research has primarily focused on using alkaline activators such as sodium hydroxide, sodium silicate, or sodium sulfate to activate single precursor materials (e.g., slag or fly ash). However, studies on composite activator systems (e.g., the synergistic effect of calcium carbide slag and sodium sulfate) and the synergistic effects of multiple solid wastes still have the following shortcomings: The hydration evolution patterns of different active geopolymers under the activation of sodium sulfate and calcium carbide slag with aging are not yet clearly understood; Environmental adaptability: Sodium sulfate is prone to causing frost heave-thaw settlement disasters in saline-alkali soils in cold and arid regions due to freeze-thaw cycles, necessitating preparatory research on the durability of solidified sulfate-contaminated soils.

For this purpose, this study used slag and fly ash as precursor materials and calcium carbide slag and sodium sulfate as activators to prepare three types of geopolymers with different activities. By analyzing the hydration process, types of hydration products, and the influence of pore structure on mechanical properties, the study investigated the mechanisms underlying the effects of different activities, curing ages, and sodium sulfate activation on the mechanical properties of these geopolymers. The research findings provide a theoretical foundation for their practical engineering applications.

## 2. Materials and methods

### 2.1. Raw materials

The calcium carbide slag used in this study is a byproduct generated during acetylene gas production by a company in Xinjiang. The slag, classified as S75 grade (Compared with benchmark cement, the 7-day activity index is ≥ 55%, and the 28-day activity index is ≥ 75%), Class F fly ash (CaO content ≤ 10%, compliant with the requirements for Class F fly ash specified in GB/T 1596–2017), Anhydrous sodium sulfate (analytical pure: purity ≥ 99%, compliant with the requirements for analytical grade reagents specified in GB/T 9853–2008) was supplied by Tianjin Zhiyuan Chemical Reagent Co., Ltd. Deionized water was used for all experiments. The cement complies with the 42.5 strength grade of Portland cement or ordinary Portland cement as per GB175–2023, while the gauge sand adheres to the Chinese ISO standard specified in GB/T17671-2021. The chemical compositions of these raw materials are detailed in **Table 1**.

**Table 1:** Chemical composition of raw materials (wt: %).

	CaO(%)	Al_2_O_3_(%)	SiO_2_(%)	SO_3_(%)	Fe_2_O_3_(%)	LOSS(%)
**Calcium carbide slag**	69.60	1.36	4.71	0.10	0.85	22.08
**Slag**	39.00	18.43	37.94	0.22	0.92	1.12
**Fly ash**	12.72	17.53	48.68	1.06	8.28	0.42

**Fig 1:**
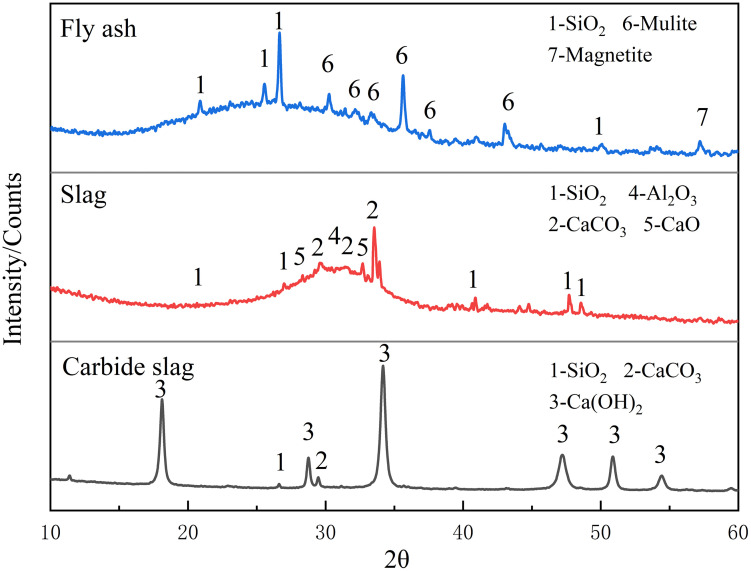
XRD patterns of calcium carbide slag, slag, and fly ash.

**Fig 2:**
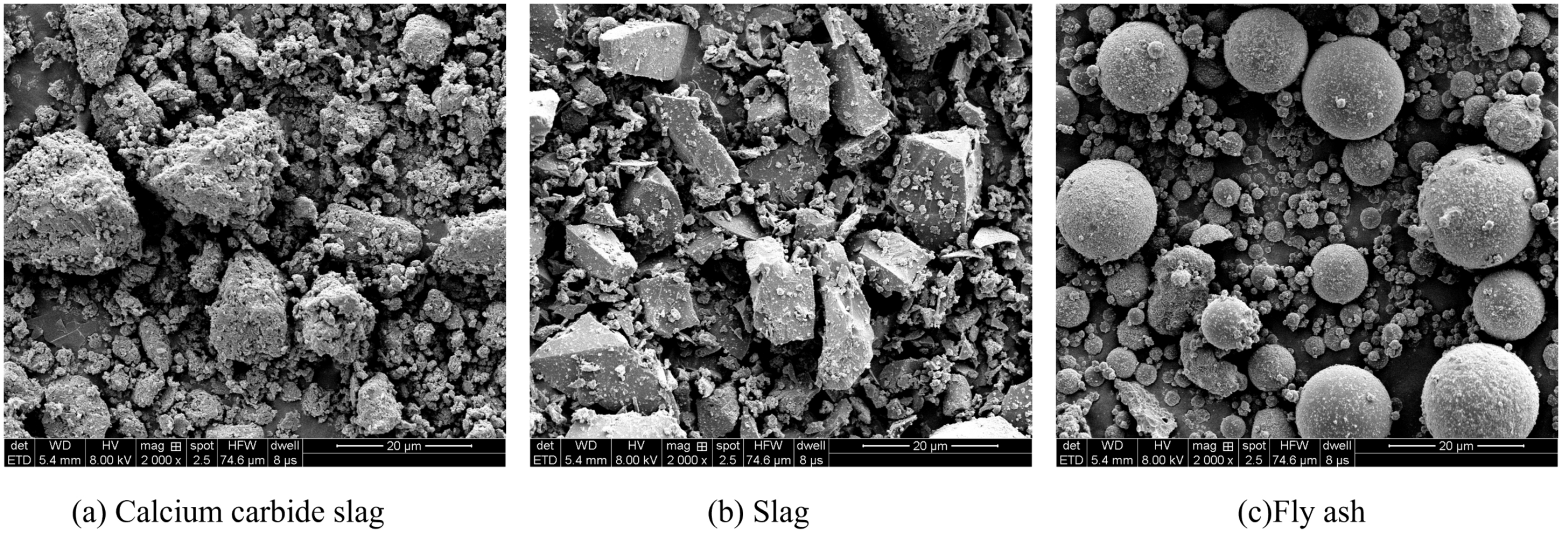
SEM images of calcium carbide slag, slag, and fly ash.

Fig 1 shows the XRD patterns of calcium carbide slag, slag, and fly ash. Calcium carbide slag is dominated by diffraction peaks of Ca(OH)_2_ and CaCO_3_. The diffraction peaks of slag and fly ash both show a hump-shaped pattern, which is typical of amorphous materials. The main diffraction peaks of slag are SiO_2_, Al_2_O_3_, CaCO_3_, and CaO. The main diffraction peaks of fly ash are SiO_2_ and mullite. Fig 2 shows the SEM images of calcium carbide slag, slag, and fly ash. It can be seen from the electron microscope images that the calcium carbide slag particles are irregular in shape, with a rough surface, a flaky or blocky structure, and a wide particle size distribution. There are a large number of pores on the surface and inside the particles [20]. The particles of slag are mostly vitreous, with smooth surfaces and spherical or irregular polyhedral shapes, and relatively small particle sizes. The particles of fly ash are mostly spherical, with smooth surfaces and relatively small particle sizes. Some fly ash particles have a honeycomb or porous structure on the surface.

In saline-alkali soils, sodium sulfate-affected soils pose the most severe hazards. Sodium sulfate undergoes a phase transition between Na_2_SO_4_·10H_2_O (mirabilite) and anhydrous Na_2_SO_4_ when exposed to temperature or humidity changes, resulting in significant volume deformation and causing the soil to expand violently, thereby compromising the stability of the roadbed. Therefore, this study uses sodium sulfate as an activator, which can react with other solid waste through hydration and also provides a theoretical basis for the subsequent solidification of sodium sulfate-salinized soil.

### 2.2. Experimental methods

#### 2.2.1. Sample preparation.

**Fig 3:**
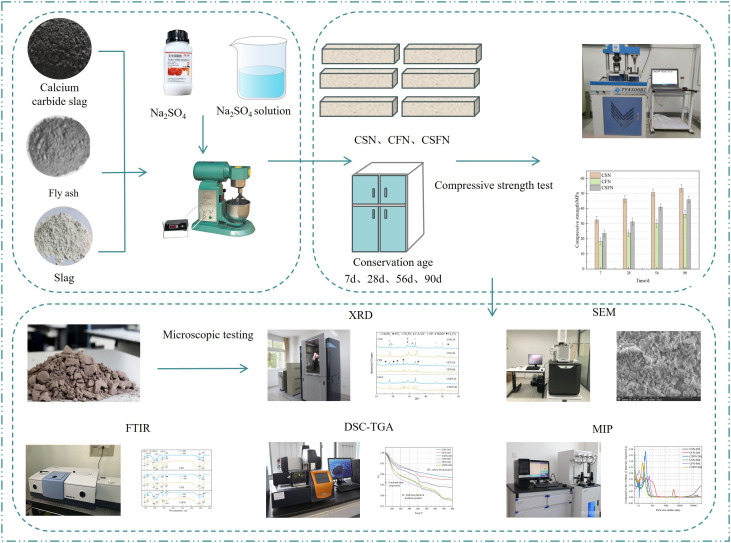
Flowchart.

Preparation of geopolymer: Prepare three types of geopolymer: calcium carbide slag-slag-sodium sulfate (CSN), calcium carbide slag-fly ash-sodium sulfate (CFN), and calcium carbide slag-slag-fly ash-sodium sulfate (CSFN). The ratio of each system is shown in **Table 2**. Prepare a sodium sulfate solution, Weigh the corresponding water and sodium sulfate powder, pour the sodium sulfate powder into the water, and stir until the sodium sulfate is completely dissolved. Weigh the raw materials, such as calcium carbide slag, fly ash, and slag, in proportion, then pour them into the planetary mixer and mix slowly for 60 s to make the raw materials evenly mixed. Continue to mix slowly for 60 s, and pour the prepared sodium sulfate solution into the mixer at a constant speed, and then mix quickly for 60 s. After the mixing is complete, pour the sample into a triple mold (40 mm × 40 mm × 160 mm) and vibrate it on a vibration table 60 times to remove the air bubbles in the geopolymer paste. After the vibration is complete, cover the mold with a layer of plastic wrap, and then demold after curing in a standard curing room (temperature 20 ± 2 °C, humidity 95%) for 24 h. Then, put the specimen back into the curing room and cure it until the corresponding age [21]. The compressive strength of the specimen is tested using a TYA-300BI compression and flexural testing machine produced by Wuxi Xinluda Instrument Equipment Co., Ltd. For the microtest specimen, after being placed in alcohol to terminate hydration, the microtest samples are removed after soaking for 1 day, placed in a 45 °C oven to dry for later use, and then subjected to subsequent tests such as XRD, FTIR, SEM-EDS, DSC-TGA, and MIP. The flowchart is shown in Fig 3.

**Table 2:** Three different cementitious material systems.

Sample Number	Sample Name	Calcium carbide slag(%)	Fly ash(%)	Slag(%)	Sulfate(%)	Curing age(d)
**1**	**CSN**	25	0	75	8	7、28、56、90
**2**	**CFN**	25	75	0	8	
**3**	**CSFN**	25	37.5	37.5	8	
**4**	**CSN2**	25	0	75	2	
**5**	**CFN2**	25	75	0	2	
**6**	**CSFN2**	25	37.5	37.5	2	
**7**	**C5SN**	5	0	75	8	
**8**	**C5FN**	5	75	0	8	
**9**	**C5SFN**	5	37.5	37.5	8	

Note: There were nine sets of tests, each containing 12 specimens. Nine different mix designs were cured, with three specimens prepared for each age for compressive strength testing. A total of 108 specimens (12 × 9) were prepared. Each set of specimens was cured at four ages: 7 days, 28 days, 56 days, and 90 days.

Due to the different activities of geopolymers, the required activation times vary. Low-activity geopolymers require longer activation times. By extending the curing time, we can study the strength changes of geopolymers with different activities and prepare for the strength changes of the three systems over a longer period of time.

Sodium sulfate is added in the form of an external admixture. CSN indicates a sample with 25% calcium carbide slag admixture, 75% slag admixture, and 8% sodium sulfate admixture; CFN indicates a sample with 25% calcium carbide slag admixture, 75% fly ash admixture, and 8% sodium sulfate admixture; CSFN indicates a sample with 25% calcium carbide slag admixture, 37.5% slag admixture, fly ash content of 37.5%, and a sodium sulfate content of 8%. CSN2 refers to a CSN sample with a sodium sulfate content of 2%, and C5SN refers to a CSN sample with a Calcium carbide slag content of 5%. The following content is the same as above.

Cement strength test: The compressive strength test method follows the cement mortar strength test method (ISO method) (GB/T 17671−2021), and the testing equipment is the TYA-300BI compressive and flexural testing machine produced by Wuxi Xinluda Instrument Equipment Co., Ltd. The compressive strength test is conducted at a rate of 2.4 kN/s. To ensure the reliability and accuracy of the test results, three parallel samples were set for each group of test specimens. The test results were considered valid if the coefficient of variation (Cv) of the same group of tests was less than 6%.

Activity index test: The compressive strength of the three systems was tested, with their activity index defined as the compressive strength ratio to cement. The raw material consumption of the activity index is shown in **Table 3**.

**Table 3:** Compares the proportions of the mortar and the test mortar.

Types of adhesive sand	Ordinary Portland cement/g	Slag/g	Fly ash/g	Chinese ISO scale sand/g	Water/ml
**Cement**	450	—	—	1350	225
**Slag**	225	225	—	1350	225
**Fly ash**	225	—	225	1350	225
**Slag + Fly ash**	225	112.5	112.5	1350	225

#### 2.2.2. Testing.

SEM-EDS test: This research employed a Thermo Fisher Scientific Quattro S field emission environmental scanning electron microscope to analyze the sample’s micro-morphology, coupled with EDS spectrum analysis to characterize the observed hydration products.

XRD test: This study used an X-ray diffractometer produced by Bruker, model D8 Advance. The scanning angle was 10° to 60°, and the scanning speed was 10°/min.

FTIR test: This study utilized a Vertex 70 infrared spectrometer manufactured by Bruker, Germany. The spectral analysis was conducted within a range of 400 cm^-1^ to 4000 cm^-1^, with samples prepared using the KBr pellet method.

DSC-TGA test: This study utilized an SDT 650 simultaneous thermal analyzer manufactured by TA Instruments. The experiments were conducted within a temperature range from room temperature to 800°C at a heating rate of 10°C/min, with nitrogen employed as a protective atmosphere.

MIP test: This research employed an AutoPore IV 9620 fully automatic mercury intrusion instrument. The pressure range for testing spanned from 0.10 psia to 61000 psia.

## 3. Results and analysis

### 3.1. Analysis of compressive strength development

**Fig 4:**
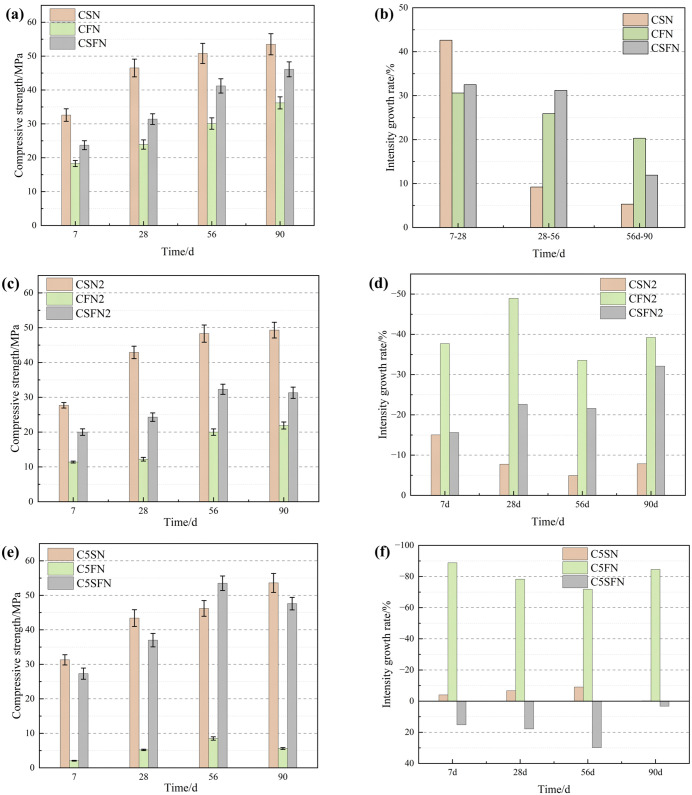
Strength properties of three geopolymers as a function of age. ((a) Patterns of intensity change with age; (b) Intensity growth rate; (c) Compressive strength of CSN2, CFN2, and CSFN2; (d) Strength growth rates of CSN2, CFN2, and CSFN2; (e) Compressive strength of C5SN, C5FN, and C5SFN; (f) Strength growth rate of C5SN, C5FN, and C5SFN).

This study focuses on three geopolymer systems with varying activity levels, synthesized from solid waste, to investigate the evolution of compressive strength at different curing ages, as illustrated in Fig 4.

Combining the data from Fig 4(b), the strength growth rate of the CSN system from 7 to 28 days is 42.6%. However, as the curing age extends to 56 and 90 days, the strength growth rate declines significantly, reaching only 9.2% and 5.3%, respectively. As shown in Fig 4(a), the CSN system exhibits the highest compressive strength, achieving 32.6 MPa at 7 days. With increasing curing time, the compressive strength rises to 46.5 MPa at 28 days, 50.8 MPa at 56 days, and 53.5 MPa at 90 days. For the CSFN system, the compressive strengths of the test blocks at 7, 28, 56, and 90 days were 3.7 MPa, 31.4 MPa, 41.2 MPa, and 46.1 MPa, respectively. The strength growth rates were calculated for different stages: 32.5% from 7 to 28 days, 31.2% from 28 to 56 days, and 11.9% from 56 to 90 days. These results indicate that the strength growth rate decreased progressively with increasing curing time. At 7 and 28 days, the compressive strength of the CSFN system was lower than the weighted average of the CSN and CFN systems. However, after 56 days, the compressive strength of the CSFN system exceeded the average of the CSN and CFN systems.

Among the systems examined, the CFN system exhibited the lowest compressive strength. At 7 days, the compressive strength was 18.3 MPa, which increased to 23.9 MPa by 28 days, representing a 30.6% increase from the 7-day strength. As the curing period extended, the compressive strength further increased to 30.1 MPa at 56 days and 36.2 MPa at 90 days, corresponding to increases of 25.9% and 20.3% compared to the previous measurement intervals, respectively. Overall, the CFN system demonstrated the highest rate of strength gain.

To further investigate the effects of salt content and calcium carbide slag on the mechanical properties of the three geopolymers, this study modified the CSN, CFN, and CSFN geopolymer systems by incorporating 2% salt and 5% calcium carbide slag, respectively. The experimental results are presented in Fig 4.

When the salt content was reduced from 8% to 2%, the compressive strengths of the CSN2 system at 7, 28, 65, and 90 days were 27.7 MPa, 42.9 MPa, 48.3 MPa, and 49.3 MPa, respectively. These values were 15.03%, 7.74%, 4.92%, and 7.85% lower than those of the original CSN system. For the CFN2 system, the compressive strengths were 11.4 MPa, 12.2 MPa, 20 MPa, and 21.9 MPa, representing decreases of 37.7%, 48.95%, 33.55%, and 39.22% compared to the original CFN system. The CSFN2 system exhibited compressive strengths of 20 MPa, 24.3 MPa, 32.3 MPa, and 31.3 MPa, which were 15.61%, 22.61%, 21.6%, and 32.1% lower than those of the original CSFN system.

These results indicate that reducing the sodium sulfate content had the most significant impact on the strength of the CFN system, suggesting a higher dependence of this system on sodium sulfate. In contrast, the CSN system exhibited relatively minor strength changes, implying a lower dependence on sodium sulfate.

To further elucidate the influence of calcium carbide slag content on the mechanical properties of the geopolymer systems, Fig 4(a) and Fig 4(e) are compared. The results show that reducing the calcium carbide slag content has varying effects on the compressive strength of the three systems over time. For the CSN system, the compressive strength decreased by 3.99%, 6.67%, 9.06%, and 0.19% at 7, 28, 56, and 90 days, respectively. In contrast, the CFN system exhibited more pronounced reductions in strength: 88.79% at 7 days, 78.24% at 28 days, 71.76% at 56 days, and 84.53% at 90 days. Conversely, the CSFN system showed an increase in compressive strength when the calcium carbide slag content was reduced: 15.19% at 7 days, 17.83% at 28 days, 29.85% at 56 days, and 3.25% at 90 days. These findings indicate that the CFN system is particularly sensitive to changes in calcium carbide slag content, with significant strength reductions observed when the calcium carbide slag content is decreased to 5%.

The development of compressive strength in geopolymer materials across three systems is closely related to the precursor activity index and the type and content of activator used. The activity indices of the three systems are shown in **Table 4**.

**Table 4:** Activity of slag and fly ash.

	Slag content(%)	Fly ash content(%)	Cement content(%)	Compressive strength(MPa)	Activity index(%)
Slag	50	—	50	46.03	91.9
Fly ash	—	50	50	34.14	68.1
Slag + Fly ash	25	25	50	40.12	80.09

The compressive strength of geopolymers increases with age, and a higher precursor activity index generally correlates with greater compressive strength. The CSN system, characterized by a high precursor activity index, exhibits rapid strength gain in the early stages, quickly reaching a high compressive strength. However, the rate of strength increase decreases significantly in the later stages and tends to level off. In contrast, the CFN system has a low precursor activity index, resulting in slow early strength gain and overall lower strength. The strength increase in the later stages is relatively high, indicating a greater dependence on accelerators such as sodium sulfate and calcium carbide slag. Notably, calcium carbide slag has a more significant effect on strength development compared to sodium sulfate. The CSFN system shows a phased strength development pattern. In the early stages (7 and 28 days), its compressive strength is lower than the weighted average of the CSN and CFN systems. However, after 56 days, the compressive strength exceeds the average of the CSN and CFN systems. The strength growth rate from 7 to 28 days is relatively high, and the rate from 28 to 56 days is the highest among the three systems. After 56 days, the growth rate decreases. Before 28 days, strength development is primarily driven by slag. From 28 to 56 days, both slag and fly ash contribute to strength gain, while after 56 days, fly ash becomes the dominant contributor. When the content of sodium sulfate and calcium carbide slag is reduced, the CSN system shows the least dependence on the activator, while the CFN system is most dependent on it. In the CSFN system, sodium sulfate exhibits a better activating effect than calcium carbide slag, indicating a higher potential for strength growth. In summary, the strength development of geopolymer materials is closely related to the activity of their precursors, the type of activator used, and its content. The CSN system demonstrates high early strength but limited late strength growth potential. The CFN system relies heavily on the activation effect of the activator, with low early strength but significant late strength development. The CSFN system combines these characteristics, exhibiting phased strength growth and achieving high late strength.

### 3.2. Phase analysis of hydration products

**Fig 5:**
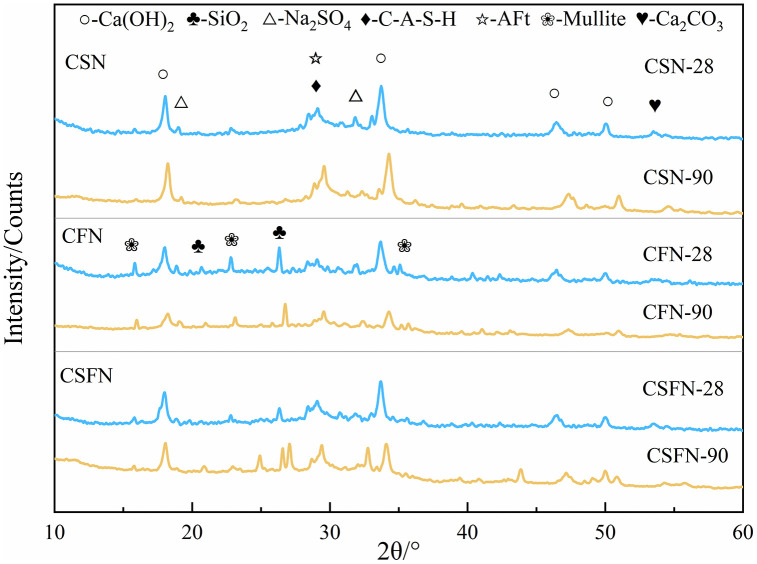
The XRD pattern of the sample.

The XRD patterns of geopolymers prepared from three different reactive precursors at 28 days of curing age are shown in Fig 5. As shown in the figure, the three systems primarily consist of C-(A)-S-H, AFt, SiO_2_, Ca(OH)_2_, and Na_2_SO_4_ [22,23]. At 28 days, the diffraction peak of Ca(OH)_2_ is narrow and high, indicating that the hydration products contain a large amount of well-crystallized Ca(OH)_2_, suggesting that the content of calcium carbide slag can meet the alkaline activation requirements of the geopolymer and maintain the alkalinity of the pore solution [24]. The detection of Na_2_SO_4_ diffraction peaks confirms the presence of unreacted sodium sulfate in the system, which also contributes to the activation effect. The basic composition of hydration products in different geopolymer systems is similar, primarily consisting of C-(A)-S-H gel and AFt, among others. At 28 days, the higher the precursor activity index, the greater the amount of C-(A)-S-H gel and AFt hydration products generated in the geopolymer (CSN > CSFN > CFN), and the lower the SiO_2_ content. This indicates that in geopolymers derived from high-activity-index precursors, the hydration reaction proceeds more thoroughly.

In the diffraction pattern of the CFN system, there is Mullite (a mineral composed of aluminosilicate) [25,26]. Compared with the XRD pattern of the raw materials, it is consistent with the mullite in fly ash. The system without fly ash does not have obvious mullite diffraction peaks, which indicates that the mullite in CFN is caused by the incomplete hydration of fly ash.

As the aging period increases, the amounts of the hydration product C-(A)-S-H and the expansive product AFt in the three-component geopolymer system increase, indicating that the hydration process of the system continues to progress. Comparing the peak intensities of hydration products at different ages in the three-component geopolymer systems, it is observed that the CFN system exhibits the greatest increase in hydration products, while the CSN system shows a relatively smaller increase. This suggests that geopolymer systems with higher precursor activity exhibit faster hydration rates in the early stages, resulting in the production of a large amount of hydration products. As the age increases, the hydration rate slows down, leading to fewer hydration products being generated in the later stages. Geopolymer systems with lower precursor activity exhibit slower hydration rates in the early stages, resulting in smaller amounts of hydration products; however, as the age increases, hydration products continue to increase at a certain rate. This shows the same trend as intensity.

### 3.3. Microscopic analysis of the topography of a geopolymer

To elucidate the mechanism by which the microstructure of the samples influences their macroscopic properties, the surface microtopography of geopolymers with different active sites was characterized. Based on the results of the SEM analysis, and through elemental analysis of the three geopolymers with different active sites using EDS, the types of hydration products were identified according to their known compositional ratios.

**Fig 6:**
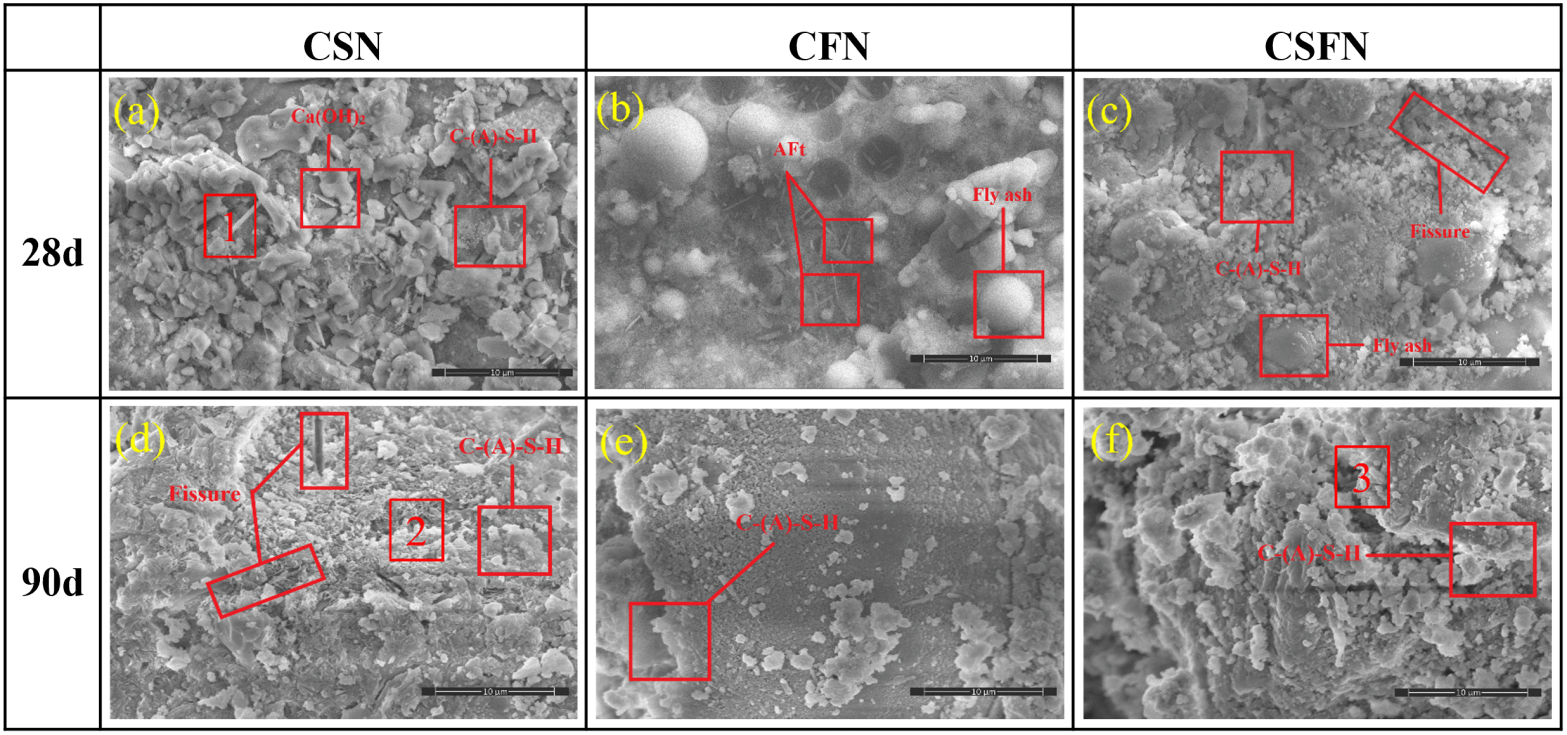
SEM of three geopolymers at different ages.

In the SEM analysis of the CSN system Fig 6(a), flaky Ca(OH)_2_ and flocculent and needle-like hydration products were observed. No unhydrated slag particles or large cracks were detected. EDS analysis (**Table 5**) confirmed that the flocculent products were C-(A)-S-H gels and the needle-like products were AFt. These hydration products were abundant and interwoven, filling the pores between the particles. Additionally, Na_2_SO_4_ crystals were observed adhering to the surfaces of unhydrated slag particles. In the 28-day CFN system, unhydrated spherical fly ash particles were observed, with flocculent C-(A)-S-H gels and needle-like AFt attached to their surfaces. This indicates that slight etching of the glassy phase on the fly ash surface had begun [27]. However, the presence of numerous unreacted Na_2_SO_4_ crystals and expansive AFt products, encapsulated on the surfaces of unhydrated particles, inhibits further hydration reactions [28], thereby limiting strength development. In the 28-day CSFN system, unhydrated fly ash particles were evident, with flocculent hydration products covering their surfaces. Granular Na_2_SO_4_ crystals and needle-like AFt were also observed. The absence of obvious pores suggests a relatively dense structure. This indicates that the synergistic effect of slag and fly ash in the CSFN system is more pronounced than in the CFN system.

**Table 5:** Atomic fractions of the characteristic point elements in Fig 6.

Element	C(%)	O(%)	Na(%)	Al(%)	Si(%)	S(%)	Ca(%)	Description
Point 1	3.81	62.38	8.09	0.7	3.21	2.16	19.65	AFt
Point 2	13.17	54.36	2.81	3.02	7.9	2.47	14.37	AFt、C-(A)-S-H
Point 3	19.84	54.84	1.76	5.33	6.8	0.92	6.91	C-(A)-S-H

As the curing age increases, the internal structure of the CSN system becomes progressively more compact. This is primarily attributed to the continuous hydration of slag in the presence of OH^-^ and SO_4_^2-^ ions. During this process, the active silicon and aluminum components within the slag are further activated and combine with Ca^2+^ ions in the system to form substantial quantities of the cementitious product C-(A)-S-H [29,30]. These hydration products coalesce within the structure, forming larger particle aggregates and stronger interconnections. They effectively fill the interparticle and interaggregate pores, creating a dense network structure. However, internally formed expansive products such as AFt and gypsum create pressure between particles. This results in an expansive and loosely connected pore structure, which may cause the compressive strength of CSN systems to slow down at longer curing ages. In the CFN system at 90 days, no distinct spherical fly ash particles were observed. The glassy phase of the fly ash gradually dissolved during the curing process, releasing reactive silicon and aluminum components into the system. These components participated in the hydration reactions in the strongly alkaline environment, resulting in the formation of significant quantities of hydration products. The hydrated products, such as Aft, are continuously formed within the pores and overlap with adjacent particles [31]. This process leads to the development of a dense network structure, thereby endowing the specimen with high strength.

At 28 days, the CSN is well hydrated and contains no unhydrated slag particles. The hydration products form a dense aggregate of fine particles with minimal gaps and cracks. In contrast, the CFN and CSFN systems still contain unhydrated, intact fly ash particles at this stage. In the CSN system, the hydration products have a needle-like and flocculent structure and relatively poor crystallinity. In the CSFN system, the hydration products change from needle-like and flocculent structures to a more granular structure. After 90 days, the CSN continues to hydrate and forms a cohesive overall structure. However, the CFN and CSFN systems remain granular and do not form a similar cohesive whole.

### 3.4. FTIR

**Fig 7:**
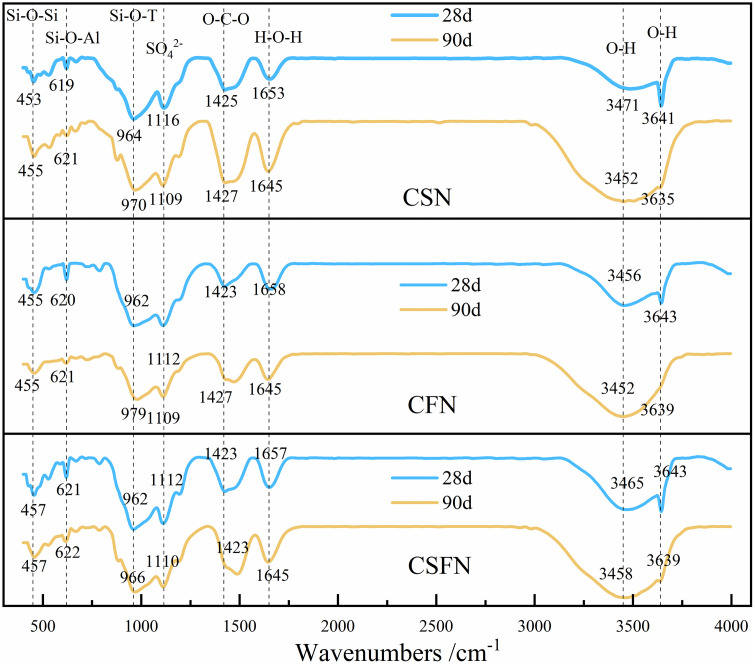
FTIR spectrum of the geopolymer.

Fig 7 presents the FTIR spectra of the three geopolymer systems. FTIR analysis was used to examine the molecular structures and functional groups within these geopolymer systems. The infrared spectra in the range of 450 cm^-1^ to 820 cm^-1^ correspond to the symmetric stretching vibrations of the Si-O-Si and Si-O-Al bonds in the silica-oxygen and aluminium -oxygen tetrahedra, which are characteristic peaks of geopolymers [32,33]. At 28 days, no significant changes were observed in the absorption peaks of these bonds across the three systems. However, as the curing period extended, the symmetric stretching vibrations of Si-O-Si and Si-O-Al gradually increased. This indicates that the geopolymer network structure becomes more dense over time, driven by the aging process. This structural evolution enhances the mechanical properties and durability of the geopolymers. The peaks in the range of 820 cm^-1^ to 1200 cm^-1^ are attributed to the asymmetric stretching vibrations of the Si-O-T bond (where T represents tetrahedral Si or Al). These peaks are characteristic of amorphous silicate minerals, such as calcium aluminates and ettringite, which are hydration products of the geopolymer reaction [34,35]. In all three geopolymer systems (CSN, CFN, and CSFN), the asymmetric stretching vibrations of the Si-O-T bond exhibited slight increases over a 90-day curing period. Specifically, the characteristic peak of the CSN system shifted from 964 cm^-1^ to 970 cm^-1^; the CFN system shifted from 962 cm^-1^ to 979 cm^-1^; and the CSFN system shifted from 962 cm^-1^ to 966 cm^-1^. This upward shift in the absorption bands indicates an increase in the polymerization degree of the aluminosilicate hydrate ions, suggesting that aging promotes further polymerization and growth of the hydration products [36,37]. This observation aligns with the continuous optimization of geopolymer structure during long-term curing.

It is also important to note that the peak near 1120 cm^-1^ is not related to the shift of the asymmetric stretching vibration peak of Si-O-T, but rather to a new asymmetric stretching vibration of SO_4_^2-^ [38]. SO_4_^2-^ can replace SiO_4_^2-^ in C-A-S-H, and the free SiO_4_^2-^ accelerates the dissolution of reactive alumina. As shown in the figure, at 28 days, the absorption peak in this range is low and narrow for the CSN system, high and wide for the CFN system, and intermediate for the CSFN system. This indicates that the hydration products in the CSN system exhibit good crystallinity and a high degree of polymerization, while those in the CFN system have poor crystallinity and a low degree of polymerization. This suggests that slag is more active, with faster hydration and polymerization of SiO_2_ and Al_2_O_3_, resulting in a greater formation of silicate gels and ettringite. This is consistent with the results of the strength tests. With increasing hydration age, the absorption peaks of all three systems increased by 90 days, with the CFN system showing the most significant increase. This indicates that the quantity of hydration products increases with age, and the CFN system maintains a relatively high growth rate in the later stages.

The absorption peak near 1420 cm^-1^ is attributed to the stretching vibration of the O-C-O bond in the CO_3_^2-^ group and is indicative of the carbonation of Ca(OH)_2_ within the geopolymer [39]. The absorption peak near 3640 cm^-1^ is associated with the O-H bond vibration in Ca(OH)_2_.

The absorption peaks near 1650 cm^-1^ and in the range of 3650 cm^-1^ to 3590 cm^-1^ are attributed to the vibrations of hydroxyl groups, indicating the presence of bound water during the hydration process of geopolymers [40]. At 90 days, the absorption peaks in the CFN and CSFN systems were found to weaken, suggesting that the pore water within the geopolymer structure is progressively reduced as the material transitions into a more gel-like product.

### 3.5. TG-DSC analysis

**Fig 8:**
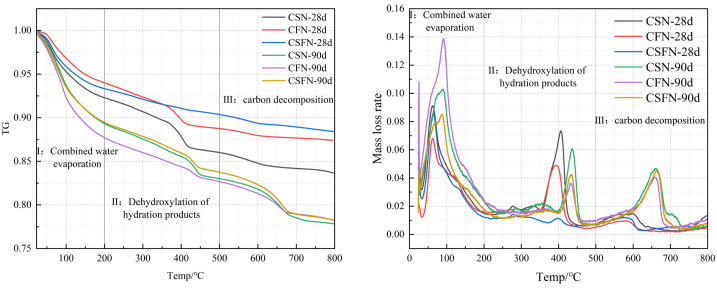
TG-DSC curve of the geopolymer.

To study the changes in the rate of hydration reactions and the amount of hydration products generated over time in different active geopolymers, thermal analysis was performed on three geopolymers cured for 28 days and 90 days, as shown in Fig 8. The weight loss below 105°C is attributed to the evaporation of free water and the decomposition of ettringite. The weight loss between 105°C and 200°C corresponds to the removal of structural water from the C-(A)-S-H gel. The weight loss between 220°C and 320°C is associated with the removal of structural water from mullite. The weight loss between 400°C and 500°C is due to the dehydration of Ca(OH)_2_, while the weight loss between 600°C and 700°C is caused by the thermal decomposition of CaCO_3_ [41]. The mass loss of the hydration products is primarily due to the dehydration of C-(A)-S-H and AFt, as well as the decomposition of Ca(OH)_2_ and CaCO_3_. The quantitative analysis of the cumulative mass loss is presented in **Table 6**.

**Table 6:** Characteristic parameters of 28-day thermal weight loss and their corresponding compressive strength.

Specimens	Total weight loss rate of C-(A)-S-H/%	Total weight loss rate of AFt/%	Weight loss rate of Ca(OH)_2_/%	Weight loss rate of CaCO_3_/%	Compressive strength/MPa
**CSN-28d**	7.6	4.9	1.8	0.6	46.50
**CSFN-28d**	6.7	4.1	1.1	0.4	20
**CFN-28d**	6.1	2.8	0.7	0.3	23.9
**CSN-90d**	10.7	6.5	2.5	3.3	53.5
**CSFN-90d**	10.6	6.6	2.2	3.4	46.1
**CFN-90d**	12.3	7.8	1.7	2.4	36.2

At 28 days, the mass loss of the hydration product C-(A)-S-H in the CSN, CSFN, and CFN systems was approximately 7.6%, 6.7%, and 6.1%, respectively, while the mass loss of AFt was about 4.9%, 4.1%, and 2.8%, respectively. These results indicate that higher geopolymer activity correlates with increased formation of C-(A)-S-H and AFt, with the order being CSN > CSFN > CFN. This is consistent with the XRD analysis results described earlier.

As the curing age increased, the mass loss of all three systems significantly increased. At 90 days, the mass loss of C-(A)-S-H in the CSN, CSFN, and CFN systems was about 10.7%, 10.6%, and 12.3%, respectively, while the mass loss of AFt was approximately 6.5%, 6.6%, and 7.8%, respectively. At this stage, the CFN system produced the most hydration products, while the CSN system produced fewer hydration products than CFN [42]. This suggests that, with increasing age, the activity of fly ash is gradually enhanced by the presence of calcium carbide slag and sodium sulfate, leading to the formation of more hydration products.

However, slag exhibits high activity in the early stages, resulting in intense hydration reactions and limited further growth in the later stages. Consequently, the CSN system, which contains slag, produces fewer hydration products than CFN. Meanwhile, the slow early hydration of CFN results in structural damage due to sodium sulfate crystals, leading to a compressive strength lower than that of CSN. Nevertheless, as the curing age continues to increase, the ongoing reaction of fly ash leads to the formation of additional hydration products, which fill structural pores and enhance the compactness of the material [43].

Calcium hydroxide decomposes into CaO and H_2_O upon heating, with 74 g of Ca(OH)_2_ yielding 18 g of H_2_O [44]. The content of calcium hydroxide was determined using thermogravimetric analysis, as shown in **Table 6**. At 28 days, the CSN system exhibited the highest percentage loss of Ca(OH)_2_, indicating that it consumed the most calcium carbide slag in the early stages. This also reflects that the hydration reaction within the calcium carbide slag-sodium sulfate system was most intense at 28 days, with the fastest hydration rate and the highest formation of gelling products (C-(A)-S-H) and expansive products (AFt). This is consistent with the results of the cumulative mass loss analysis. In contrast, the CFN system had the lowest percentage loss, indicating a slower hydration rate and minimal formation of gelling and expansive products. The CSFN system’s hydration rate remained intermediate between the two, aligning with previous analyses. As the curing age increased, the consumption of Ca(OH)_2_ in the three systems showed that CSFN > CSN > CFN, with minimal overall differences. In the CFN system, the Ca(OH)_2_ content increased from 0.7% to 1.7% with age, indicating that the activity of fly ash was progressively stimulated, leading to ongoing hydration and increased consumption of Ca(OH)_2_. This finding is consistent with the previous cumulative mass loss analysis results.

### 3.6. Pore structure

**Fig 9:**
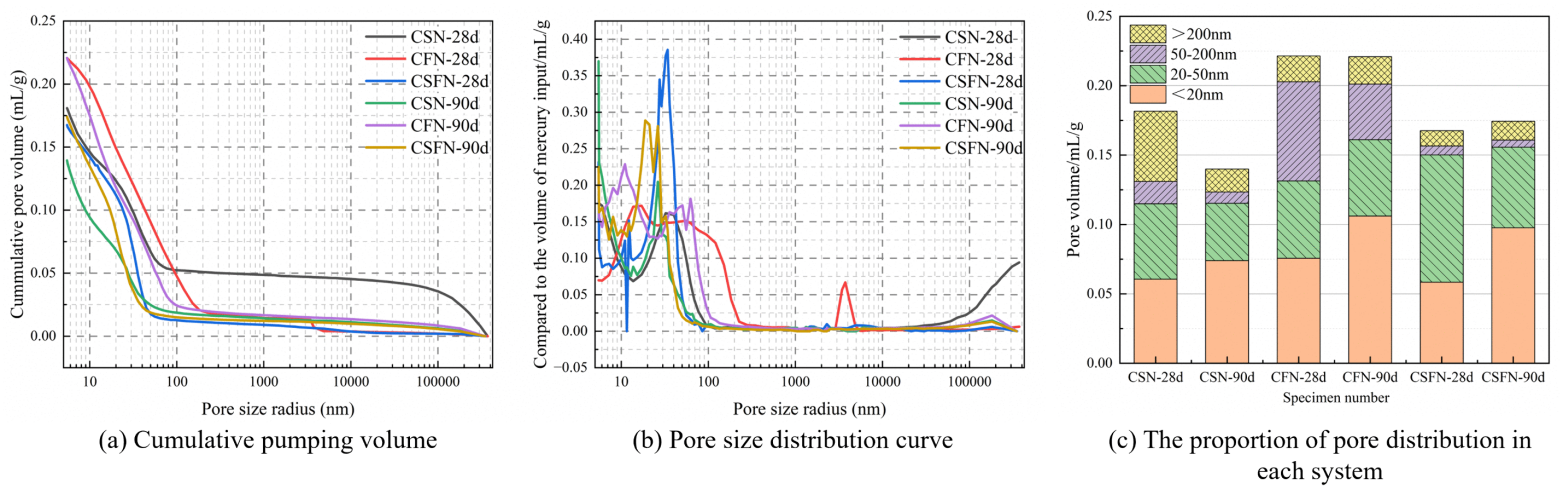
28d-90d Changes in pore structure of three geopolymers.

Fig 9 shows the mercury intrusion data for the CSN and CFN systems, further analyzing the internal pore changes of the samples at different curing ages. As the curing age increases from 28 d to 90 d, the overall cumulative mercury intrusion volume of the CSN sample decreases, the pore size above 20,000 nm decreases significantly, and the pore size between 20 and 40 nm increases slightly. This indicates that in the CSN system, the internal structure is optimized mainly in two ways: One is the micro-aggregate effect of slag powder particles, and the other is the generation of a large amount of hydration products by slag under the dual stimulation of calcium carbide slag and sodium sulfate to fill the pores of the system and improve the strength of the sample. Therefore, in the early stage of the system, the slag powder particles can fill the large pores to play the role of a micro-aggregate. At the same time, the hydration products of CSN-28d cementitious materials cement with each other in the pores to fill the pores together, thereby strengthening the compressive strength of the sample. As the age increases, the internal structure of the CSN system continues to optimize. At this time, the slag reaction is more complete, the role of the micro-aggregate decreases, and the hydration reaction is mainly relied on to generate a large amount of hydration products such as C-(A)-S-H, AFt, and CaCO_3_, which intertwine with each other in the pores, making the internal structure more compact, thereby improving the compressive strength of the sample, which is consistent with the strength law described above. For CFN specimens at the 28-day age, large pores appear at 3000–6000 nm. The early CFN system has more pores larger than 100 nm than the CSN system, so the compressive strength of CFN is also lower. Although the early CFN specimen also improves the internal structure of the system in two ways, fly ash has a large number of large pores in the early stage due to its internal glass phase. The hydration at room temperature and atmospheric pressure was slow, so there were many large pores in the system. As the age of the sample increased, the fly ash continued to depolymerize under the dual stimulation of calcium carbide slag and sodium sulfate. The reactive Si and Al elements in the fly ash participated in the hydration reaction, and the hydration products continued to increase, making the structure more compact and the compressive strength higher, which was consistent with the law of compressive strength.

Based on their impact on geopolymer properties, pores are categorized into four types: harmless (<20 nm), less harmful (20–100 nm), harmful (100–200 nm), and more harmful (>200 nm). Fig 9(c) shows the distribution of pore volume in different active geopolymers. At the 28-day age, the lower the activity, the larger the total pore volume, which is not conducive to strength development. CSN-28d has more harmful pores, which may be because the early slag has high activity, generating a large amount of hydration products, too much AFt, which is stacked in the structure and expands the internal pores, resulting in more harmful pores. The CFN-28d has the most total pores because the fly ash has the lowest activity, and in the early stages there are also a large number of unhydrated spherical fly ash particles, which loosen the internal structure, resulting in the most harmful pores and harmful pores. The total porosity of CSFN-28d is less than that of the other two systems, and it has the fewest harmful pores, harmful pores, and its internal structure is more compact. Therefore, the simultaneous addition of slag and fly ash is beneficial to optimize the internal pore structure. As the curing age increases from 28 d to 90 d, the harmless pores of the three different active polymer systems all increase. This indicates that with the increase of age, the hydration products have a strong synergistic effect inside the pores, significantly optimizing the pore structure and making the block more dense. As the age increases, the pore volume of the CSN system decreases significantly, the internal structure becomes denser, and the compressive strength increases, which is consistent with the previous analysis results. CFN-90d as the age increases, the overall pore structure does not change much, but its harmful pores decrease sharply and its harmless pores increase. Combined with the previous analysis, because the fly ash is less reactive and the hydration reaction is slow, the internal structure is relatively loose, resulting in low compressive strength. As the age increases, its internal harmful pores decrease significantly, and the harmless pores increase, which is the reason for the linear growth of its compressive strength. CSFN-90d as the age increases, the harmless pores increase, the harmful pores decrease, and the internal structure becomes denser, resulting in a significant increase in compressive strength, which is consistent with the previous analysis results.

## 4. Discussion

**Fig 10:**
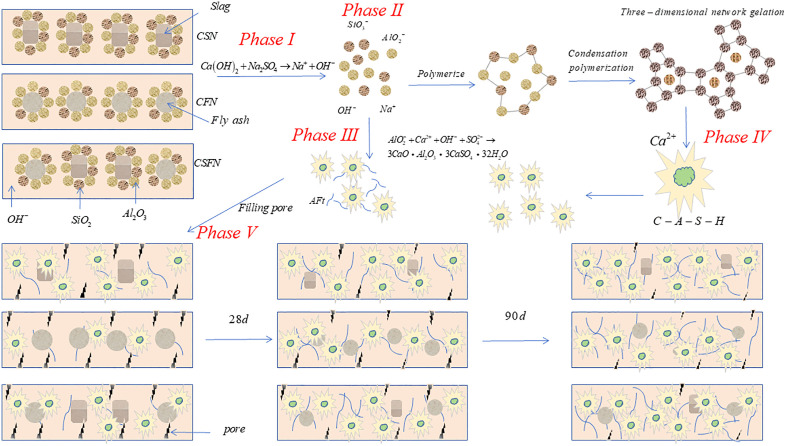
Mechanism of hydration.


To further elucidate the reaction processes and hydration mechanisms of the three geopolymer systems, this section summarizes the aforementioned analysis and presents a schematic diagram of the hydration mechanism, as shown in Fig 10. Based on the preceding analysis, we classify the hydration reaction into five stages: the first stage involves the directed dissolution of calcium carbide slag to form Ca(OH)_2_, while Na_2_SO₄ reacts with Ca(OH)_2_ in the liquid phase to form a strong base, providing an alkaline environment for the system (1). The second stage primarily involves the hydrolysis reaction between slag and fly ash. The hydration reaction rate in this stage is correlated with the activity indices of the three systems. Slag has the highest activity, with its internal active silicon and aluminum being more easily activated. Fly ash has the lowest activity, with its own active silicon and aluminum being more difficult to activate, thus requiring increased curing time and activators for activation. In the third stage, Ca(OH)_2_ from the calcium carbide slag continuously supplies Ca^2+^ ions to Na_2_SO_4_, reacting with the gel on the surface of fly ash particles or slag particles and dissolved Al_2_O_3_ in the liquid phase to form the hydration product AFt. In the fourth stage, the hydration and polymerization reactions of the geopolymer can be fully activated by strong alkali, increasing the free [SiO_4_]^4-^ and [AlO_4_]^5-^ ions in the reaction system, thereby generating more C-(A)-S-H gel (2). The fifth stage involves the gel product C-(A)-S-H bonding with Aft to fill the pores of the geopolymer, forming a dense network structure, thereby enhancing the strength of the system by filling the pores. As shown in the figure, the black waves represent the pores of the sample, with the size and number of waves indicating the size and quantity of the pores. The blue lines represent the hydration product Aft, and the flocculent products in the figure are C-(A)-S-H gels. The most active CSN produced the most hydration products at 28 days, with fewer pores; the least active CFN geopolymer hydrates more slowly, producing fewer hydration products and more pores. As the curing age increases, the increase in hydration products for the most active CSN is not as significant as at 28 days; however, CFN’s hydration products continue to increase, pores gradually decrease, and strength improves; CSFN’s hydration products gradually increase, pores gradually decrease, and the increase in strength remains between the two systems.

$$Na_{\text{2}}SO_{\text{4}}+Ca{\left(OH\right)}_{\text{2}}+2H_{\text{2}}O\to CaSO_{\text{4}}\cdot 2H_{\text{2}}O↓+2NaOH$$ (1)

$$Ca{\left(OH\right)}_{2}+Al_{2}O_{3}+H_{2}O\to CaO\cdot Al_{2}O_{3}\cdot SiO_{2}\cdot H_{2}O$$ (2)

To further elucidate the quantitative relationship between influencing factors and the compressive strength development of solid waste-based geopolymer, an empirical equation was established based on experimental results [45]. The proposed model comprehensively considers the effects of curing age, activity index, sodium sulfate dosage, and calcium carbide residue dosage, reflecting their combined contribution to the strength evolution process.


$$f=3.53+1.083\left[8.27\times 10^{-5}A^{0.22}{\left(B+0.68C+0.09D\right)}^{2.65}e^{\left(1.36\times 10^{-5}AB\right)}\right]$$ (3)

**Fig 11:**
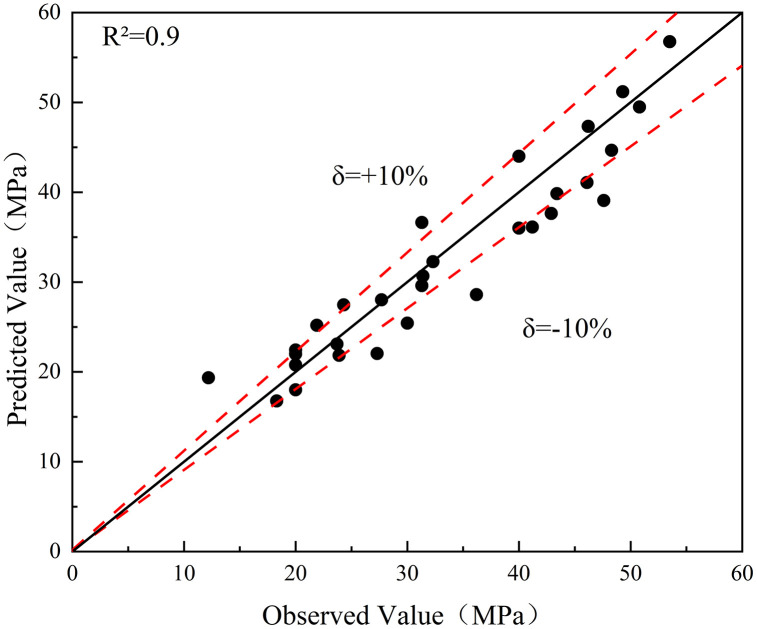
Comparison between predicted and observed compressive strength of solid waste-based geopolymer.

In the equation, *f* represents compressive strength (MPa); A denotes age (d); B is the activity index; C is sodium sulfate content (%); D is calcium carbide residue content (%). This equation indicates that compressive strength increases with curing time and activator activity, while appropriate additions of sodium sulfate and calcium carbide residue further promote hydration product formation. The exponential term reflects synergistic enhancement between curing time and activity index. The model’s predicted results show good agreement with experimental data, with data points generally distributed close to a straight line of y = x, yielding R² = 0.9 and an error range within ±10%. This indicates the model’s reliability. This empirical model was developed based on experimental data from solid waste-based geopolymers co-activated with slag and fly ash. Therefore, its applicability is primarily confined to similar alkali-activated systems where the activator composition and curing temperature are comparable to those in this study. Within this scope, the equation reliably predicts the compressive strength development of solid waste-based geopolymer across different curing ages and activator ratios. It provides a quantitative reference for mix design and performance prediction in engineering applications. The comparison results between the predicted compressive strength and the measured compressive strength of solid waste-based geopolymer are shown in Fig 11.

The Ca(OH)_2_ in the calcium carbide slag continuously provides Ca ions for the Na_2_SO_4_, which reacts with the gel trapped on the surface of the fly ash particles or slag particles and the AlO_2_ dissolved in the liquid phase to form calcium aluminate sulfate hydrate Aft, as shown in Equation (4). Some of the calcium aluminate sulfate hydrate can also react with gypsum to form Aft, as shown in Equation (5).


$$Al{O_{2}}^{-}+Ca^{2+}+OH^{-}+S{O_{4}}^{2-}\to 3CaO\cdot Al_{2}O_{3}\cdot 3CaSO_{4}\cdot 32H_{2}O$$ (4)

$$3CaO\cdot Al_{2}O_{3}\cdot 6H_{2}O+3\;CaSO_{4}\cdot 2H_{2}O+20H_{2}O\to 3CaO\cdot Al_{2}O3\cdot 3CaSO_{3}\cdot 32H_{2}O$$ (5)

**Fig 12:**
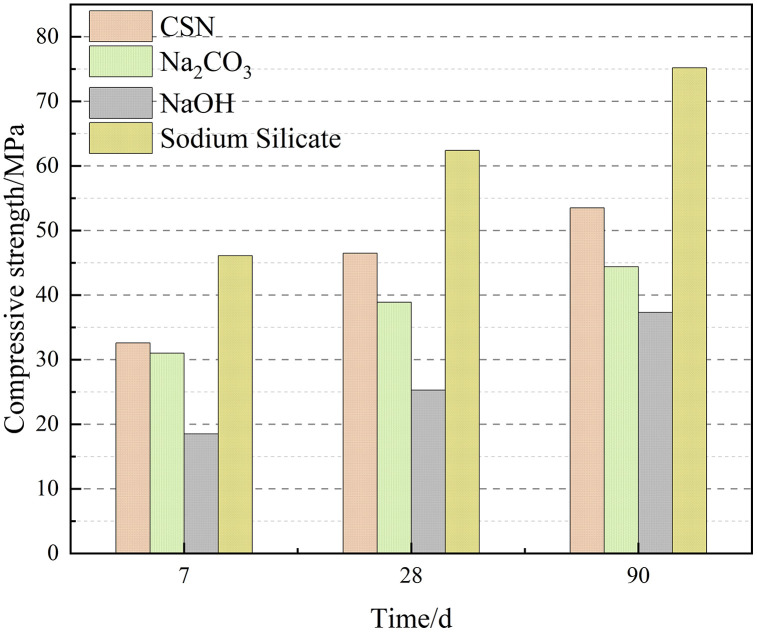
Effect of Different Activators on Compressive Strength Development in Slag Systems.

This study systematically compared the effects of four activators—sodium sulfate (CSN), sodium carbonate, sodium hydroxide, and water glass—on the compressive strength development of slag-based geopolymers [46–51]. Results indicate that the reaction mechanisms of different activator systems significantly influence both the early reaction rate and the subsequent structural densification process of geopolymers. The effect of different activators on the development process of compressive strength in slag systems is shown in Fig 12.

Regarding early strength development, the CSN system achieved 32.6 MPa at 7 days, significantly higher than the 18.5 MPa of the NaOH system and slightly exceeding the 31 MPa of the Na_2_CO_3_ system, demonstrating superior early activation capability. This stems from the simultaneous provision of Na⁺ and SO_4_^2-^ ions in the CSN solution. This system not only enhances leaching of Ca, Al, and Si from slag by increasing alkalinity but also facilitates the formation of multi-phase gel structures—including C-(A)-S-H and calcium aluminate hydrates—via SO_4_^2-^ ion introduction, thereby strengthening structural integrity and early strength. Despite higher alkalinity, the NaOH system’s monobasic environment inhibits sulfur-containing alumina phase formation, resulting in lower early strength.

As curing age increases, the strength development trends of each system diverge further: the Na_2_CO_3_ system achieves only 38.9 MPa at 28 days, indicating its late-stage strength development remains constrained by insufficient reacting environment alkalinity. Conversely, the CSN system reaches 46.5 MPa at the same age and increases to 53.5 MPa at 90 days, demonstrating its ability to sustain activation reactions and promote continuous gel phase growth over the long term. In contrast, although the NaOH system showed improvement in the later stage (reaching 37.3 MPa at 90 days), its overall polymerization degree remained limited.

The water glass system achieved 46.1 MPa at 7 days, demonstrating the most outstanding early strength. However, its strength gain slowed significantly after 28 days (increasing from 62.4 MPa at 28 days to 75.2 MPa at 90 days), indicating the reaction rapidly reached silica-alumina saturation, limiting later densification potential. In contrast, the CSN system, while exhibiting slightly lower early strength, demonstrated more stable reaction persistence and higher late-stage densification potential, reflecting a favorable balance between early and long-term performance.

From an engineering and environmental perspective, the CSN system not only achieves significant strength development but also effectively immobilizes free SO_4_^2-^ ions in contaminated soils, suppressing salt swelling and secondary crystallization, thereby exhibiting high environmental compatibility. In contrast, while the water glass system offers higher strength, its production process generates approximately five times the carbon emissions of the CSN system and incurs higher economic costs. Consequently, the CSN activation system demonstrates significant comprehensive advantages in balancing mechanical performance, environmental sustainability, and soil remediation potential.

## 5. Conclusion

1. The geopolymer’s strength development shows a clear dependence on precursor activity: higher activity index yields greater early strength but reduces late-stage strength potential. Specifically, the highly active CSN system demonstrates excellent early strength yet limited later growth; the less active CFN system displays lower early strength but remarkable late strength increase; while the CSFN system, benefiting from the synergistic interaction between slag and fly ash, achieves both staged strength development and superior later strength.
2. The activation effects of calcium carbide slag and sodium sulfate significantly influence the strength development of geopolymers, though with system-dependent variations. While the CSN system shows relatively low activator dependence, the CFN system demonstrates the highest sensitivity to activators, with calcium carbide slag exhibiting more pronounced effects than sodium sulfate. Notably, in the CSFN system, sodium sulfate displays superior activation performance compared to calcium carbide slag, indicating its greater potential for enhancing strength development.
3. The synergistic activation effect of calcium carbide slag and sodium sulfate in the fly ash-slag blended system significantly optimized both the phase composition and microstructural evolution of hydration products. This dual-activation mechanism not only facilitated high early-age compressive strength but also ensured continuous strength development with prolonged curing. The above performance characteristics indicate that the CSFN system is particularly suitable for the production of prefabricated components, while the CFN system is more suitable for scenarios that require low early strength but long-term stability.
